# The impact of job burnout on public service motivation and the moderating role of perceived organizational support: a survey among resident work team members in Chinese villages

**DOI:** 10.3389/fpsyg.2026.1859652

**Published:** 2026-08-12

**Authors:** Zhe Zhu, Leyi Shao, Heran Zou, Wanqiang Xu, Yilong Sheng

**Affiliations:** 1 School of Management, Wuhan Institute of Technology, Wuhan, Hubei, China; 2 Enterprise and Environment Coordinated Development Research Center of Hubei Province, Wuhan, Hubei, China; 3 College of Public Administration, Huazhong University of Science and Technology, Wuhan, Hubei, China

**Keywords:** job burnout, moderating role, perceived organizational support, public service motivation, resident work team members

## Abstract

Since 2013, the Chinese government has promoted rural revitalization by deploying resident work team members to villages. As policy implementers within the governance framework of China, resident work team members’ levels of public service motivation (PSM) influence the strategic outcomes of rural revitalization. Employing hierarchical regression analyses and moderation tests, this study provides an empirical examination of resident work team members in the Wuling Mountains region of China. The findings indicate that the overall level of PSM among resident work team members is high, yet job burnout is present to a considerable extent. Job burnout is negatively associated with resident work team members’ PSM, and perceived organizational support (POS) moderates this association. At higher levels of POS, the negative relationship between job burnout and PSM is significantly attenuated. Policy implications underscore the need to address resident work team members’ job burnout, strengthen their professional capacity development, standardize the assessment and operational standards for village work, and improve accountability mechanisms, welfare provisions, as well as the alignment of promotion and placement arrangements, thereby fostering an organizational climate that encourages innovation. This study enriches and extends the antecedents and moderating factors of PSM theory, offering theoretical support and policy guidance for realizing China’s rural revitalization strategy and for establishing a comprehensive, multi-level talent-support system for rural development.

## 1 Introduction

To address rural poverty and achieve comprehensive rural revitalization, China has deployed more than 3 million cadres from party and government organs as well as from public institutions to participate in village-based support work. The embedded governance model embodied by resident work team members has effectively improved rural public services and grassroots governance, contributing to the decisive victory in poverty alleviation. Under current standards, 98.99 million rural poor people have been lifted out of poverty, and 832 impoverished counties have been removed from the list. To further consolidate and expand poverty alleviation gains, promote agricultural and rural modernization, and realize rural revitalization, the Central Committee has decided to continue dispatching resident work teams to rural areas. By 2025, the nation will still host 255,000 resident work teams and more than 750,000 team members rooted in the fields.

Resident work team members, as policy implementers within China’s governance framework, constitute a pivotal force in advancing rural revitalization. Their public service actions directly determine the strategic outcomes. Yet human behavior is driven by motivation, and for resident work team members embedded at the periphery of the governance system, their behavioral logic likewise follows this principle. Public Service Motivation (PSM) denotes the altruistic tendency of government employees to be driven by organizational goals; as a specific psychological orientation, it refers to the inner drive to act in accordance with public values, characterized by altruism, dedication, and commitment to public value, predominantly attracted by public institutions and organizational missions (Zeng, 2008; Perry and Wise, 1990). It is also a key determinant of public service behavior, shaping government employees’ altruistic and prosocial actions (Homberg et al., 2019). Consequently, as the motivational source prompting resident work team members to shoulder responsibility, the level of PSM directly influences their behavioral choices and work effectiveness in grassroots settings. To enhance incentives for resident work team members to perform in basic public service delivery, it is essential to further stimulate their PSM. Elevating PSM not only improves job performance (Bellé, 2013), but also promotes the alignment of grassroots cadres’ career aspirations with the ethos of “serving the people,” achieving a deeper congruence between individual career objectives and the organization’s public mission, thereby optimizing grassroots governance and public service delivery and facilitating policy implementation and reform (Liu and Chen, 2019).

At present, advancing rural revitalization has become a central national priority in the mission of realizing the great rejuvenation of the Chinese nation. In this context, sustained village support work plays a vital role. However, as village work deepens, some grassroots cadres have exhibited burnout (Zhou, 2023; Fang and Wu, 2020). Burnout is a psychosocial state characterized by emotional exhaustion, depersonalization, and a reduced sense of achievement arising from role stress; it represents a comprehensive psychological response that is observable across occupations (Schaufeli et al., 2001). Existing evidence indicates that burnout substantially diminishes public sector work engagement and organizational performance and affects altruistic motivation (Chen and Xiao, 2020; Harrison, 1983), though burnout is not intractable; social support and organizational interventions can alleviate burnout (Wang et al., 2015). Accordingly, the pertinent questions arise: what are the levels of burnout and PSM among resident work team members in Chinses villages? Does burnout influence resident work team members’ PSM? How can the adverse effects of burnout be mitigated? These questions demand rigorous empirical inquiry.

**Figure 1:**
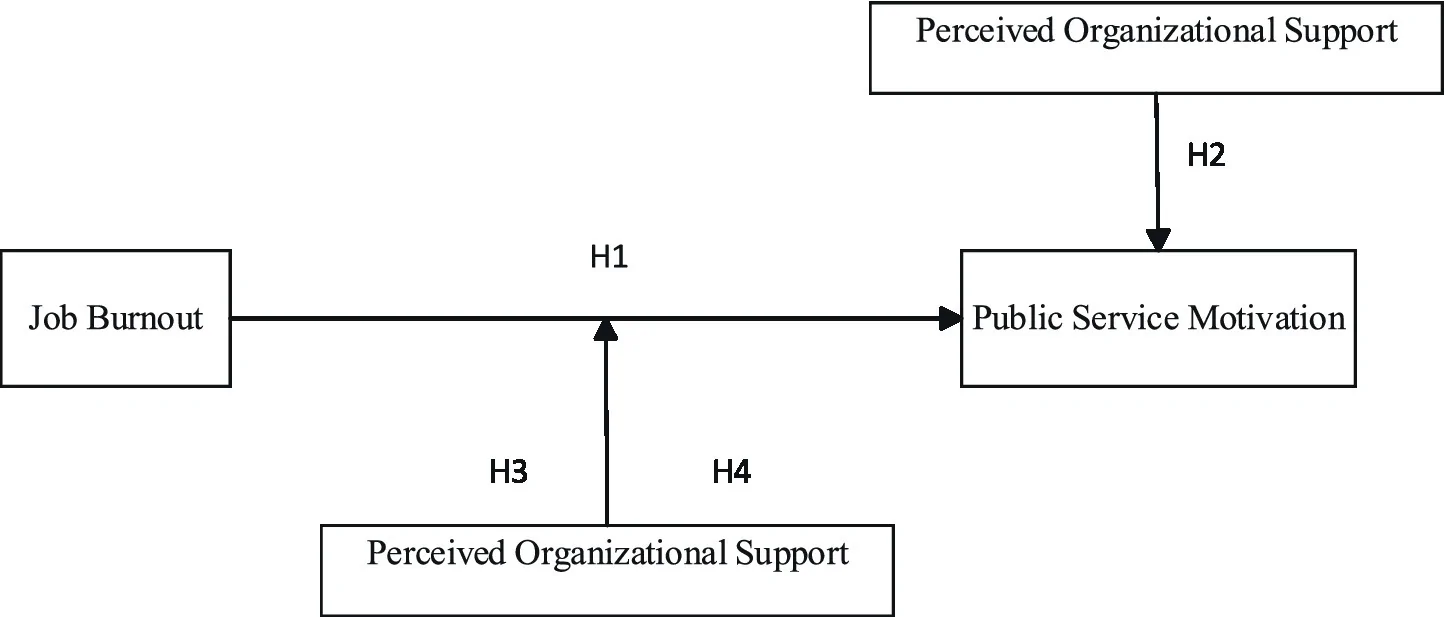
The hypothesized model.

The remainder of this paper is structured as follows. First, we review the relevant literature and develop the research hypotheses across three domains: the relationship between job burnout and public service motivation among resident work team members; the association between public service motivation and perceived organizational support; and the interactive mechanisms linking job burnout, public service motivation, and perceived organizational support. Next, we outline the research design, including the sampling strategy, data collection procedures, and analytical methods used in the study. We then present the empirical results based on survey data collected from resident work team members in the Wuling Mountains region. Finally, we discuss the theoretical and practical implications of the findings, offer policy guidance for realizing China’s rural revitalization strategy and for establishing a comprehensive, multi-level talent-support system for rural development (Figure 1).

## 2 Literature review and research hypothesis

### 2.1 Job burnout and public service motivation among resident work team members

The concept of job burnout was first proposed by the American psychologist Freudenberger, and is also referred to as “occupational burnout” and “job exhaustion” (Freudenberger, 1974). Job burnout refers to a state of physical and mental exhaustion resulting from prolonged and high-intensity work pressure. When individual competence does not effectively match job demands, job burnout may occur and seriously undermine individuals’ willingness to work and their performance.

At present, with the continuous advancement of performance targets and assessment tasks associated with the rural revitalization agenda, the scope of resident work team members’ responsibilities has expanded markedly, while the complexity of their work and the intensity of task-related pressure have increased in tandem. In the course of promoting “strong villages and prosperous residents” and enhancing grassroots governance capacity, governance distortions such as “trace-based management” and bureaucratic formalism have become increasingly salient. Consequently, some cadres find themselves compelled to devote substantial time and effort to responding to inspections, supervision, and compliance checks at multiple administrative levels, rather than concentrating on the substantive work of rural development (Yang and Gao, 2025).

Within the institutional setting of rural revitalization, resident work team members also confront notable tensions stemming from an inverted configuration of authority and responsibility. On the one hand, the relatively closed dynamics of the “township–village” relationship may heighten the likelihood of friction and conflict between resident work team members and township or village officials in day-to-day implementation. On the other hand, a “dual-track” governance arrangement can engender a relationship with village Party branches and village committees that is simultaneously cooperative and competitive, thereby shaping—at least to some extent—the strength and effectiveness of rural revitalization performance outcomes (Li and Zhang, 2021). Against this backdrop, a subset of village-resident cadres may exhibit reduced engagement or even a degree of role detachment from rural affairs. Meanwhile, work fatigue and occupational burnout have become increasingly evident among grassroots village-resident cadres (Zhou, 2023; Hao, 2022).

The occurrence of job burnout significantly reduces individual job satisfaction and increases their turnover intention (Galanis et al., 2025). The characteristic dimensions of emotional exhaustion, depersonalization, and low personal sense of achievement are negatively correlated with individual job satisfaction (Wu et al., 2009). Job burnout may also contribute to civil servants’ passive behavior—often described as “doing nothing for the government”—leading to a loss of work enthusiasm and a gradual decline in job satisfaction (Wen and Zhang, 2019).

Existing studies have also found a significant negative relationship between job burnout and job identity (Angelini, 2023). Sowislo and Orth (2013), in their trauma model, proposed that an increase in job burnout would undermine individuals’ job identity. High professional stress negatively affects individuals’ career well-being through the chain mediating effects of job burnout and occupational identity (Wang et al., 2014). Job burnout, manifested as work fatigue and exhaustion, directly undermines professional identity and reduces individuals’ altruistic motives (Liu, 2016; Rayner et al., 2018). PSM, as an altruistic tendency driven by organizational goals, is influenced by several antecedents 1, including individual characteristics, professional identity, and job satisfaction (Liu, 2019). Civil servants’ levels of PSM correspond to their professional identity and job satisfaction (Zhao and Gao, 2014). At the same time, professional identity also significantly affects the level of PSM (Chen et al., 2018; Kim et al., 2015). Red tape leads to a decline in individuals’ professional identity, thereby reducing their PSM (Moynihan and Pandey, 2007). Taken together, the above literature indicates that the relationship between job burnout and PSM among resident work team members can be understood as a role-based process of motivational erosion. Although PSM has traditionally been defined as a relatively stable personal value orientation and professional ethic, established through early socialization processes and the formation of public-service-related values, and mainly referring to individuals’ enduring commitment to public values, its expression may nevertheless change in response to situational variations. That is, individuals’ willingness to engage in public service and the extent to which such willingness is translated into behavior may vary across specific work environments. Specifically, in the context of China’s rural revitalization, although village-based work is politically mandated and job burnout does not fundamentally alter the stable trait component of PSM among resident work team members, prolonged village residency, increasingly heavy work tasks, and concerns about individual career development may still affect their PSM. Under the high-pressure task environment of grassroots governance, job burnout may weaken the situated expression of PSM and its capacity for behavioral translation by depleting psychological resources, undermining role identification, and reducing self-efficacy. Based on the above theoretical analysis, we put forward the research hypothesis:

*Hypothesis 1* Job burnout is negatively associated with the PSM of resident work team members.

### 2.2 Public service motivation and perceived organizational support for resident work team members

Perceived organizational support (POS) theory posits that when individuals perceive care, assistance, and recognition from their organization in both work and life domains, their level of work engagement increases substantially. Consequently, employees’ willingness to contribute to the organization largely depends on the extent to which they perceive organizational support (Eisenberger et al., 1986). Prior research suggests that higher work engagement is positively associated with stronger PSM. POS not only exerts a significant negative effect on turnover intention but also reduces employees’ willingness to seek job changes (Ahmed et al., 2015). Existing studies further indicate that enhanced POS is linked to greater work engagement, lower turnover intention, and stronger organizational commitment. Grounded in social exchange theory, employees who perceive greater organizational support tend to demonstrate higher organizational loyalty, stronger organizational commitment, and increased work involvement, which collectively stimulate their willingness to exert effort toward organizational goals (Li et al., 2022). Enhanced POS strengthens affective commitment, fostering employees’ willingness to contribute to organizational values and objectives while reducing the likelihood of continuance commitment (Eisenberger et al., 2001). Empirical evidence also shows that organizational commitment significantly enhances PSM among grassroots civil servants, thereby improving their work performance (Ma and Mei, 2017). Moreover, organizational commitment mediates the relationship between network centrality and PSM: employees positioned centrally in organizational networks tend to exhibit stronger commitment and, in turn, higher levels of PSM (Zhang, 2018). Under high-commitment work systems, organizational commitment exerts a pronounced positive effect on PSM among grassroots cadres (Liu and Chen, 2019).

Within the context of China’s rural revitalization strategy, the duties of village-based officials—such as rural governance and grassroots organizational development—are inherently public service–oriented and driven by PSM. Support from the dispatching organizations enables these officials to secure project resources for targeted villages, thereby improving local public service capacity (Xu and Li, 2017). Compared with employees in relatively stable organizational settings, resident work team members operate within a temporary, task-oriented, and cross-boundary bureaucratic arrangement. Their POS is therefore likely to be shaped not only by care and recognition from the dispatching organization, but also by broader institutional supports, including policy authorization, resource coordination, assessment alignment, accountability protection, welfare guarantees, and career-related arrangements across multiple levels of rural governance (Wang et al., 2019). Organizational support directly shapes the work willingness and assistance outcomes of village work teams; insufficient support often results in stagnation, whereas robust support improves performance and fosters positive recognition from local communities (Zhou, 2023).

Furthermore, prior studies reveal that increased POS significantly enhances organizational citizenship behavior (Zhang et al., 2025), particularly altruistic behavior (Susskind et al., 2000). Employees who perceive stronger organizational support tend to reciprocate with more extensive public service behaviors. POS is also strongly associated with motivational enhancement; motivation, as a fundamental internal driver of behavior, positively shapes employees’ organizational actions (Li et al., 2024). In this study, POS refers primarily to enabling and resource-based support, including organizational recognition, care, assistance, value affirmation, and practical support in dealing with work difficulties. It is therefore conceptually different from excessive administrative intervention, intensified supervision, or politically driven accountability pressure. Based on the above literature, the following hypothesis is proposed:

*Hypothesis 2* POS is positively associated with resident work team members’ PSM.

### 2.3 Job burnout, public service motivation, and perceived organizational support for resident work team members

Extensive empirical research has confirmed the detrimental consequences of job burnout on individuals. Resources obtained through organizational and social support—such as opportunities for promotion—can significantly mitigate burnout, indicating that increased work resources exert a positive psychological motivational effect on employees (Chen and Wang, 2019). Organizational support not only reduces work fatigue among frontline staff (Liu and Fan, 2017) but also attenuates the negative impact of surface acting on job satisfaction. Moreover, POS plays a significant moderating role in the relationship between work fatigue and job satisfaction (Wen and Hou, 2018). Prior studies further argue that effective improvements in employees’ work attitudes and occupational cognition require strengthened organizational support and heightened levels of POS (Organ and Ryan, 1995).

Since the 20th National Congress of the Communist Party of China, village-based officials have been entrusted with new mission-oriented responsibilities, accompanied by increasing work complexity and intensity. Elevated work involvement often limits their ability to balance work and family roles, thereby intensifying work–family conflict and work stress (Chen and Zuo, 2018). Under persistent role stress, some grassroots cadres exhibit symptoms of burnout, including physical and emotional exhaustion and diminished sense of accomplishment (Shu and Liu, 2019), which negatively affect their willingness to provide public services in rural areas and lead to issues such as unstable career commitment and poor work performance (Ye, 2016).

Although a significant relationship exists between work involvement and occupational stress, studies have shown that high POS strengthens the positive effect of mission valence on grassroots civil servants. Additional evidence suggests that while increased work stress elevates burnout, social support is negatively correlated with each dimension of burnout. Social support enhances employees’ sense of security and organizational belonging, which in turn stimulates intrinsic motivation by buffering job burnout (Zhou, 2023). Furthermore, POS moderates the relationship between relational leadership behaviors and PSM, with stronger effects observed under high POS. Civil servants in supportive organizational environments tend to interpret leadership support more positively, thereby strengthening work enthusiasm and increasing work engagement through heightened PSM (Chen and Lin, 2016). Based on the above analysis, the following hypotheses are proposed:

*Hypothesis 3* POS moderates the negative association between job burnout and resident work team members’ PSM.

*Hypothesis 4* Higher levels of POS attenuate the negative association between job burnout and PSM; that is, under higher POS, the inverse relationship between job burnout and PSM becomes less pronounced.

## 3 Method

### 3.1 Samples and data collection

This study adopted a multi-stage field survey procedure to collect data from resident work team members in the Wuling Mountain Area of China. First, the Wuling Mountain Area was selected as the research region because it is a typical site for examining resident work team members under the rural revitalization strategy. The region is located at the junction of Guizhou, Hubei, Hunan, and Chongqing, has a large ethnic minority population, and continues to face important tasks related to consolidating poverty alleviation outcomes and improving rural governance (Research Center for Development and Utilization of Characteristic Resources in Wuling Mountain Area, 2016). Second, the target respondents were clearly defined as resident work team members who were dispatched by Party and government agencies, public institutions, enterprises, or mass organizations and who were directly engaged in village-based assistance and rural revitalization work. Third, questionnaires were distributed to eligible respondents through field investigation and coordination with local administrative departments. Participation was voluntary and anonymous, and respondents were informed of the academic purpose of the survey before completing the questionnaire. A total of 400 questionnaires were distributed to resident work team members in Chinese villages. Among them, 378 questionnaires were returned, yielding a response rate of 94.5%. After excluding 72 questionnaires due to incomplete responses or invalid answering patterns, a final sample of 306 valid questionnaires was used for analysis. Among the 306 resident work team members surveyed, 233 were male (76.14%) and 73 were female (23.86%). The mean of the gender variable was 0.76 with a standard deviation of 0.43. In terms of age distribution, 256 respondents (64%) were between 31 and 60 years old, with an average age of 44 years (see Table 1).

**Table 1:** Socio-demographic statistics for samples.

Socio-demographic characteristic		Total samples *N* = 306	%
Gender	Male	233	76.14
	Female	73	23.86
Age	18–30 years	50	16.34
	31–50 years	154	50.33
	51 years and above	102	33.33
Work experience	0–5 years	48	15.69
	5–15 years	76	24.84
	16 years and above	182	59.48
Education level	High school and below	24	7.84
	Technical secondary school	25	8.17
	Associate degree	56	18.30
	Bachelor’s degree and above	201	65.69
Distance to workplace	1 h and below	128	41.83
	1-3 h	131	42.81
	3 h and above	47	15.36

### 3.2 Ethics approval and consent to participate

This study was approved by the Ethics Review Committee of the School of Management, Wuhan Institute of Technology (Approval No. 2023–226-02PJ). All methods were performed in accordance with the relevant guidelines and regulations. Informed consent was obtained from all participants prior to the survey. Participation was voluntary and anonymous.

### 3.3 Analytical method and variable measurement

#### 3.3.1 Job burnout

Job burnout was measured using a 15-item scale adapted from Li and Shi (2003) and Wang and Shu (2018). The scale includes nine positively worded items, such as “Village assignment work makes me feel physically and mentally exhausted,” “I feel worn out after finishing my daily village assignment work,” “Village assignment work is indeed very stressful for me,” “Since starting this job, I have become increasingly less interested in village assignment work,” and “I am no longer as enthusiastic about village assignment work as I used to be.” In addition, the scale contains six reverse-coded items, including “I can effectively solve problems that arise in village assignment work,” “I consider myself competent in carrying out village assignment work,” and “I feel very happy when I accomplish certain tasks in village assignment work.” Reverse-coded items were recoded during data analysis. Higher total scores indicate higher levels of job burnout among the respondents.

#### 3.3.2 Perceived Organizational Support (POS)

POS was assessed using the scale developed by Chen and Lin (2016), which consists of six items. Sample statements include: “My organization values the contributions I make in village work,” “My organization recognizes my personal goals and values,” “When encountering difficulties in village work, my organization always provides support,” and “My organization is proud of my achievements in village work.” Responses were measured on a five-point Likert scale (1 = strongly disagree, 5 = strongly agree), with higher scores indicating stronger POS.

#### 3.3.3 Public Service Motivation (PSM)

PSM was measured using the scale developed by Bao and Li (2016), which includes eight items, such as: “I am highly interested in participating in village work,” “I hope to contribute to rural socioeconomic development through village work,” “I believe impoverished populations should have equal development opportunities,” “I feel distressed when beneficiaries encounter difficulties,” and “I feel indignant when beneficiaries are treated unfairly.” The scale uses a five-point Likert scoring method, with higher scores indicating stronger PSM.

#### 3.3.4 Control variables

PSM is influenced by various individual characteristics, including gender, age, work tenure, educational level, administrative rank, and income level (Ye and Lai, 2011; Lewis and Frank, 2002; Wang and Sun, 2018; Wang and Shu, 2018). Additionally, longer village service duration and greater distance from home are associated with increased burnout among grassroots cadres, which in turn significantly affects PSM (Shu and Liu, 2019). Therefore, these variables were included as control variables in the analysis.

## 4 Analytic strategy

In the initial stage of data analysis, we first conducted reliability tests and confirmatory factor analyses to assess the internal consistency and construct validity of the key variables, including job burnout, public service motivation, and perceived organizational support. After establishing the measurement quality, we performed descriptive statistics and correlation analysis to explore preliminary relationships among the major variables and control variables. Next, hierarchical multiple regression analysis was employed to examine the associations of job burnout and perceived organizational support on public service motivation. Hierarchical multiple regression was selected because the hypotheses focus on the incremental effects of job burnout, POS, and their interaction on PSM after controlling for individual and assignment-related characteristics. This approach allows the explanatory contribution of each block of variables and the moderating effect of POS to be clearly assessed. Since the measurement properties of the constructs were first verified through reliability analysis and confirmatory factor analysis, the use of validated composite scores in regression analysis was appropriate for the purpose of hypothesis testing. Following this, we introduced interaction terms to examine the moderating role of perceived organizational support in the relationship between job burnout and public service motivation. Finally, we generated interaction plots to visualize differences in the association across varying levels of perceived organizational support, thereby providing a clearer interpretation of the moderating effect.

## 5 Results

### 5.1 Confirmatory factor analysis

The reliability and validity of the measures for job burnout, PSM, and POS were assessed using Cronbach’s *α* coefficients and confirmatory factor analysis (CFA). The results indicate that all three constructs demonstrated strong internal consistency, with Cronbach’s α values exceeding 0.90. The CFA results further show that the standardized factor loadings for all items were above 0.60, the average variance extracted (AVE) values exceeded 0.50, and the composite reliability (CR) values were greater than 0.70. These findings collectively suggest that the three constructs exhibit satisfactory convergent validity. The detailed results are summarized in Table 2.

**Table 2:** Analysis of reliability and aggregate validity.

Variable	Cronbach α	Analysis item	Standard load factor (std. Estimate)	AVE	CR
Job burnout	0.961	JOB1	0.785	0.624	0.961
		JOB2	0.803		
		JOB3	0.775		
		JOB4	0.806		
		JOB5	0.769		
		JOB6	0.850		
		JOB7	0.885		
		JOB8	0.803		
		JOB9	0.795		
		JOB10	0.742		
		JOB11	0.805		
		JOB12	0.750		
		JOB13	0.793		
		JOB14	0.793		
		JOB15	0.697		
Public service motivation	0.923	PSM1	0.795	0.600	0.923
		PSM2	0.837		
		PSM3	0.760		
		PSM4	0.794		
		PSM5	0.778		
		PSM6	0.746		
		PSM7	0.762		
		PSM8	0.741		
	
Perceived organizational support	0.949	POS1	0.890	0.754	0.948
		POS2	0.848		
		POS3	0.874		
		POS4	0.880		
		POS5	0.854		
		POS6	0.867		

Regarding the discriminant validity of PSM, job burnout, and POS, the study first applied the Fornell–Larcker criterion. The results indicate that the smallest square root of AVE among the three constructs was 0.775, which was substantially higher than the maximum inter-construct correlation coefficient of 0.226. This demonstrates strong discriminant validity among the variables. Model fit indices further support this conclusion. In the three-factor model, the ratio of chi-square to degrees of freedom (χ^2^/df) was below 3, RMSEA was less than 0.10, RMR was below 0.05, and GFI, CFI, and NFI all exceeded 0.80. These values meet commonly accepted thresholds for good model fit and outperform those of the two-factor and one-factor models. Furthermore, following the recommendations of Podsakoff et al. (2003), the Unmeasured Latent Method Construct (ULMC) approach was employed to further examine potential common method variance. A latent method factor (CMV) was added to the baseline three-factor model to create a competitive model. The results indicated that the inclusion of this latent factor did not yield a significant improvement in the model fit (ΔRMSEA = 0.008, and ΔNNFI = 0.018 all of which were well below the 0.05). These findings provide further evidence that common method bias does not substantially threaten the validity of the results and is within an acceptable range. Collectively, these findings confirm that the four constructs exhibit strong discriminant validity, and the overall reliability and validity of the measurement model are adequate for subsequent empirical analyses. Detailed results are presented in Tables 3, 4.

**Table 3:** Discrimination validity AVE square root value table.

	Job burnout	Public service motivation	Organizational support
Job burnout	0.790		
Public service motivation	−0.350	0.775	
Perceived organizational support	−0.122	0.226	0.868

**Table 4:** Model fitting analysis table.

Model	Factor	χ^2^/*df*	GFI	RMSEA	RMR	CFI	NFI	NNFI
Three factors Model + CMV	A, B, C, CMV	2.169	0.855	0.062	0.043	0.945	0.903	0.935
Three factors model	A, B, C	2.490	0.827	0.070	0.049	0.924	0.880	0.917
Two factors model	A, B + C	6.698	0.563	0.136	0.234	0.708	0.674	0.684
Single factor model	A + B + C	10.281	0.412	0.174	0.266	0.522	0.499	0.486

A: job burnout; B: public service motivation; C: perceived organizational support; CMV: Common Method Variance factor.

### 5.2 Descriptive statistic and correlations

This study employed regression-based moderation analysis to examine the effect of job burnout on resident work team members’ PSM, as well as the moderating role of POS. Prior to conducting the regression analysis, the correlations among the key variables were explored. The results indicate that job burnout is significantly and negatively correlated with PSM, POS is significantly and negatively correlated with job burnout, and POS is significantly and positively correlated with PSM. Descriptive statistics further show that the mean value of PSM was 4.528 with a standard deviation of 0.560, suggesting that resident work team members generally exhibit a high level of PSM. PSM was also found to be significantly and positively associated with age, years of work experience, and administrative rank, implying that older resident work team members, those with longer tenure, and those at higher administrative levels tend to demonstrate stronger PSM. Notably, PSM exhibited a significant negative skewness and a high kurtosis. From a governance perspective, this statistical distribution may align with the unique institutional selection and professional ethos of village-based cadres in China. As key implementers of the rural revitalization strategy, these individuals are often discussed in prior literature as possessing high political commitment and mission-driven altruism, which provides a plausible contextual background for the concentration of scores at the higher end of the PSM scale. However, correlation analysis alone cannot identify potential moderating effects; therefore, regression analysis and moderation testing were conducted for further examination (Table 5).

**Table 5:** Descriptive statistics and correlation analysis of variables.

Var	Mean	SD	Skew	Kurt	Gen	Age	WY	AL	RT	Edu	Inc	HD	JB	POS	PSM
Gen	1.239	0.427	1.233	−0.483	1										
Age	2.824	1.081	−0.396	−1.032	−0.115***	1									
WY	3.605	1.574	−0.553	−1.339	−0.128***	0.682***	1								
AL	2.111	1.234	0.631	−1.041	−0.150***	0.428***	0.369***	1							
RT	3.343	1.516	−0.243	−1.486	−0.198***	−0.085	−0.013	−0.070	1						
Edu	3.745	0.857	−0.993	1.491	−0.004	−0.204***	−0.257***	0.095	−0.134***	1					
Inc	2.944	1.225	0.257	−0.713	−0.119***	0.245***	0.244***	0.525***	−0.041	0.093	1				
HD	2.837	1.330	0.177	−1.053	−0.093	−0.205***	−0.271***	−0.109	0.169***	0.262***	−0.012	1			
JB	2.222	0.945	0.572	−0.057	0.031	−0.136***	−0.124***	−0.146***	0.124***	−0.011	−0.177***	0.130***	1		
POS	3.871	1.214	−1.332	0.736	−0.028	−0.055	−0.016	−0.047	0.080	0.008	0.025	−0.011	−0.122***	1	
PSM	4.528	0.560	−2.477	11.149	0.020	0.358***	0.350***	0.289***	−0.231***	−0.025	0.126***	−0.267***	−0.350***	0.226***	1

Var, variable; SD, standard deviation; Skew, Skewness; Kurt, Kurtosis; Gen, gender; WY, working years; AL, administrative level; RT, resident time; Edu, education level; Inc, income; HD, home distance; JB, job burnout; POS, perceived organizational support; PSM, public service motivation. ***p < 0.001, **p < 0.01, *p < 0.05.

### 5.3 Hypothesis testing

In the regression analysis for moderation testing, Model 1 incorporated the control variables and the independent variable—job burnout—to examine the main association between job burnout and resident work team members’ PSM. Model 2 added the moderating variable, POS, to assess its direct association with PSM. Model 3 further included the interaction term between job burnout and POS to evaluate the moderating role of POS in this association. To reduce potential multicollinearity, all key variables were mean-centered prior to analysis. The regression results are presented in Table 6.

**Table 6:** Hypothesis test analysis.

Variable	M 1		M 2		M 3	
	Coefficient	Standard error	Coefficient	Standard error	Coefficient	Standard error
Gen	0.050	0.067	0.056	0.064	0.033	0.063
Age	0.073**	0.036	0.081**	0.035	0.078**	0.034
WY	0.059**	0.025	0.057**	0.024	0.060**	0.023
AL	0.067**	0.028	0.075***	0.027	0.062**	0.027
RT	−0.052***	0.019	−0.060***	0.018	−0.054***	0.018
Edu	0.034	0.035	0.031	0.034	0.032	0.033
Inc	−0.037	0.026	−0.043	0.026	−0.036	0.025
HD	−0.055**	0.022	−0.052**	0.022	−0.050**	0.021
JB	−0.160***	0.030	−0.141***	0.029	−0.129***	0.028
POS			0.106***	0.022	0.109***	0.021
Job burnout*POS					0.080***	0.020
Constant	4.213***	0.225	4.221***	0.217	4.238***	0.212
*R* ^2^	0.302		0.353		0.386	
Adjustment *R*^2^	0.280		0.331		0.363	
Observations	306		306		306	

Gen, gender; WY, working years; AL, administrative level; RT, resident time; Edu, education level; Inc, income; HD, home distance; JB, job burnout; POS, perceived organizational support; PSM, public service motivation. ***p < 0.001, **p < 0.01.

**Figure 2:**
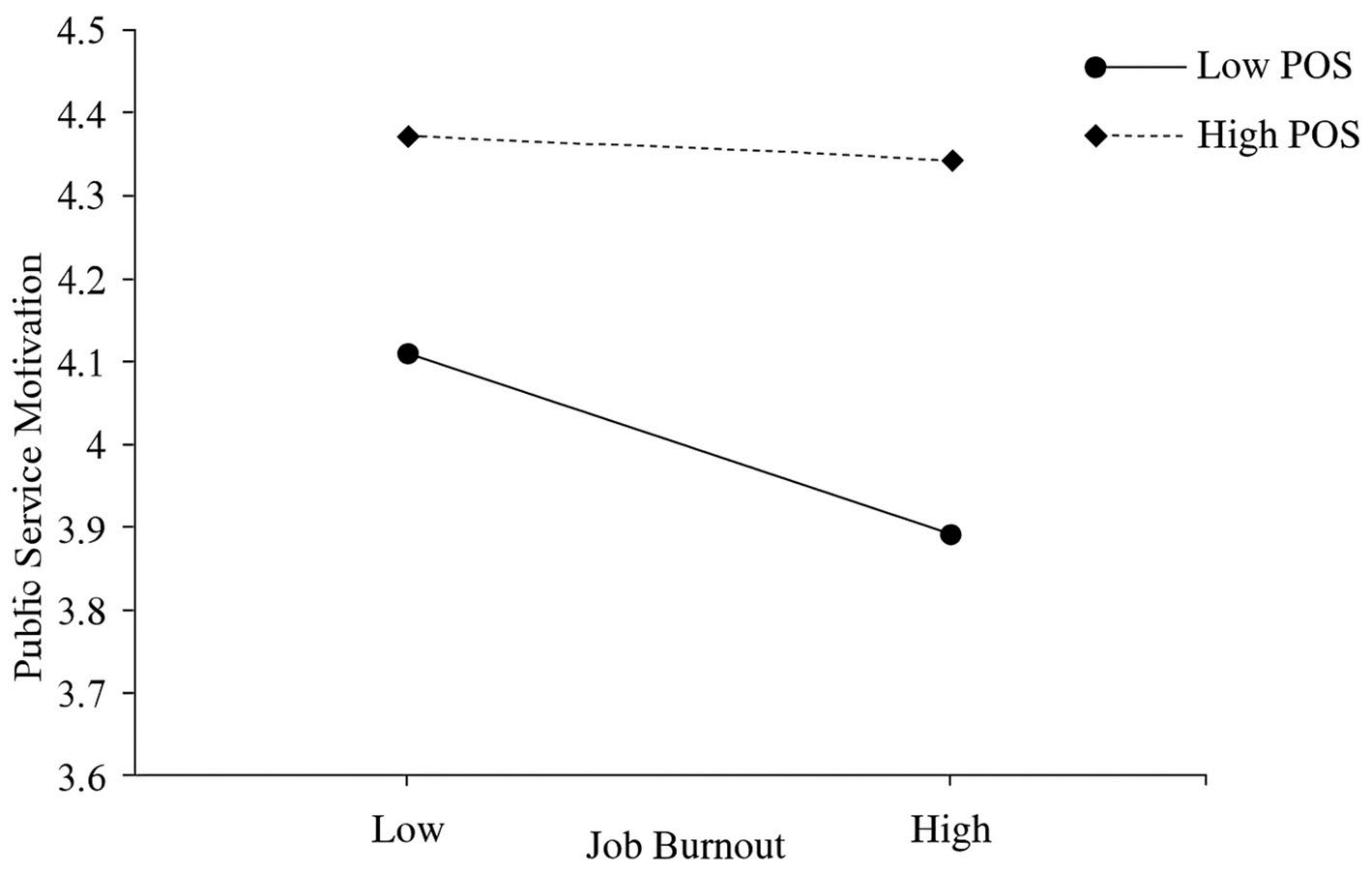
The role of POS in the relationship between job burnout and PSM of resident work team members in the village.

The results indicate that job burnout is significantly and negatively associated with resident work team members’ PSM (*β* = −0.160, *p* < 0.01), providing support for Hypothesis 1. After incorporating POS into the model, the findings show that POS is significantly and positively associated with PSM (*β* = 0.106, *p* < 0.01), thereby supporting Hypothesis 2. When the interaction term between job burnout and POS was added, a significant change in the *F*-value was observed from Model 2 to Model 3, and the interaction term itself reached statistical significance (*p* < 0.01). These results indicate that POS moderates the negative association between job burnout and PSM (*β* = 0.080, *p* < 0.01), supporting Hypothesis 3. Further analysis reveals that the association between job burnout and PSM varies across different levels of POS. Specifically, compared with conditions of low POS, the negative association between job burnout and PSM is less pronounced under high POS, supporting Hypothesis 4. The positive coefficient of the interaction term indicates that POS moderates this negative association. In other words, when resident work team members perceive higher organizational care, recognition, and resource support, the negative association between job burnout and PSM appears attenuated. The moderating pattern is illustrated in Figure 2.

## 6 Conclusions and discussion

### 6.1 Conclusions and suggestions

The findings of this study indicate that, within the ongoing process of rural revitalization, resident work team members as a whole display a relatively high level of PSM, while job burnout simultaneously presents a non-negligible concern within this group. Empirical results indicate that job burnout is negatively associated with resident work team members’ PSM, and POS moderates this association: at higher levels of POS, the negative relationship between job burnout and PSM is substantially attenuated. Drawing on these empirical associations, relevant literature, and observations of grassroots practices, the potential contextual drivers of job burnout among resident work team members may be interpreted in relation to several key factors.

First, the coexistence of multiple assessment authorities and heterogeneous assessment standards, together with inconsistencies between formal assessment criteria and practical operational needs, has significantly intensified resident work team members’ sense of occupational fatigue. At the grassroots level, resident work team members are frequently occupied with responding to inspections and evaluations conducted by different administrative tiers; assessment teams at provincial, municipal and county levels, as well as third-party evaluators, often adopt divergent standards for appraising village-based work. Each new round of inspection requires extensive revision of forms and documentation to cater to these varying requirements, resulting in a considerable amount of time and energy being consumed by repetitive data entry and document preparation. In addition, the current assessment system is characterized by an overemphasis on procedural compliance and an underemphasis on substantive outcomes, making it difficult to comprehensively evaluate actual performance. In some localities, assessment efforts focus primarily on whether documents are complete and forms fully filled in, while paying insufficient attention to regional development, policy implementation effectiveness and residents’ sense of gain. The management mechanisms for resident work team members are also underdeveloped in certain areas, with clear manifestations of fragmentation between vertical and horizontal lines of authority and inadequate managerial responsibility. Many resident work team members continue to shoulder work assigned by their dispatching organizations during their village posting, and some even take leave or temporarily depart their village position in the name of the sending unit. Meanwhile, the day-to-day management and assessment responsibilities fall largely on county (district) and township (subdistrict) departments in charge of rural revitalization, which often feel unable or unwilling—and sometimes reluctant—to supervise senior resident work team members dispatched from higher-level provincial or municipal agencies. Faced with disparate assessment requirements and complex operational standards, many grassroots poverty alleviation and resident work team members feel at a loss, leading to a heightened sense of task ambiguity and increased occupational fatigue.

Second, the high density of work tasks combined with incomplete accountability mechanisms contributes significantly to job burnout. The investigation reveals that resident work team members not only bear major responsibility for implementing the rural revitalization strategy but also undertake extensive grassroots governance functions. Long working hours and intensive schedules—often described as “5 + 4” working days or “day-and-night shifts”—have become routine. In some cases, resident work team members follow a “3 + 2” pattern, spending 3 days per week in the village and 2 days at their dispatching organization to manage their original duties, thereby intensifying role conflict and challenging their perceived role competence. The heavy workload imposes substantial physical and psychological pressure, which may exceed individuals’ coping capacity and ultimately trigger job burnout. This situation is compounded by a low degree of alignment between responsibilities and formal authority. Under an accountability system that remains incomplete, resident work team members may become risk averse, fearful of taking responsibility and reluctant to exercise initiative, gradually shifting from active “agents” to passive “data clerks.” The lack of a robust accountability framework can also foster perceptions that their hard work is neither adequately recognized nor fairly rewarded, thereby aggravating feelings of job insecurity and injustice and weakening their occupational identity and self-efficacy. Moreover, constraints stemming from limited governance resources and mismatches between authority and responsibility pervade the routine work of resident work team members, creating additional burdens. In such an environment marked by resource scarcity, imbalanced responsibilities and stringent layers of supervision, resident work team members often have very limited scope to exercise their capabilities, further reinforcing their sense of frustration and burnout.

Third, the formation of a “town–village closed loop” in governance and entrenched path dependence in traditional administrative practices also contribute to job burnout among resident work team members. The investigation reveals that, in some regions, resident work team members are constrained or even marginalized by cadres at township and village levels, with non-cooperation and conflict occurring intermittently. Path-dependent reliance on a traditional, extensive management approach—one that prioritizes major events over everyday details and outcomes over processes—has led grassroots governments to habitually adopt governance styles that impede precision, refinement, concreteness and human-centered practices. Such an orientation results in superficial governance that fails to reach sufficient depth or coverage. At its core, this problem reflects a lack of awareness regarding quantification, precision, standardization and institutionalization. Under these circumstances, resident work team members may feel tremendous pressure because they must operate under strict assessment regimes, with limited resources, while simultaneously responding to complex and evolving rural governance demands. In addition, in some localities, township and village cadres either become overly dependent on resident work team members or disengage from day-to-day responsibilities, shifting virtually all tasks and responsibilities onto them. This pattern of responsibility transfer and blame shifting exacerbates job burnout among resident work team members.

Addressing these embedded contextual drivers may require a broader array of institutional interventions rather than isolated administrative remedies. Combining these empirical patterns with prior literature and insights from field observations, several possible policy implications can be identified to provide stronger structural, resource, and psychological support for resident work team members. First, it is essential to strengthen capacity building for resident work team members. This requires establishing and improving institutional arrangements for selection, training, management, incentives, performance appraisal, protection and evaluation, and ensuring that these policies are effectively implemented. A normalized mechanism for village assistance, a mechanism for integrating financial and material resources, and corresponding incentive and assessment systems should be refined. At the same time, resident work team members’ knowledge of and commitment to rural work should be enhanced by providing systematic and periodic training on rural revitalization policies and rural governance, enabling them to better understand patterns of rural development and to learn from the experience of exemplary rural communities. Organizational support should not be limited to assistance in specific work tasks; it should also focus on cultivating job competence and local attachment. This can be achieved by inviting experienced rural practitioners and academic experts to provide targeted training and by strengthening exchanges among resident work team members, thereby improving their capacity for rural work and their overall level of professional competence.

Second, work assessment and operational standards should be unified, and the accountability mechanism for village-based work should be improved. It is necessary to further standardize the criteria for village-based work and make full use of modern technologies to optimize work processes. This will help avoid multi-headed and multi-standard assessments, reduce the burden of repetitive documentation, and alleviate work-related stress. A unified and diversified performance evaluation framework should be established, combining both rigid and flexible indicators—such as governance effectiveness, capacity to engage with residents and work initiative—to fully reflect actual performance and effectively curb formalistic tendencies such as “indicator-based revitalization” and “document-based revitalization.” At the same time, the accountability framework for resident work team members should be refined, including the establishment of mechanisms for fault tolerance and error correction so as to reduce their concerns about taking initiative. Resident work team members who exhibit perfunctory behavior, engage in corruption or falsify information in village-based work should be held strictly accountable, thereby ensuring the seriousness and integrity of village assignments. Through these measures, resident work team members can be guided and enabled to “settle down and take root,” focusing their efforts on addressing the concrete problems of target groups and promoting the sustainable development of rural areas.

Third, welfare benefits, promotion pathways and placement arrangements for resident work team members should be improved in an integrated manner. It is necessary to further enhance resident work team members’ welfare and to strengthen top-level design of promotion and post-assignment mechanisms. By introducing supporting measures to raise remuneration and establish a reasonable wage growth mechanism, resident work team members’ incomes can be aligned with their workload and contributions, allowing the valence and expectation of promotion to jointly serve as effective motivational drivers. For resident work team members who have made outstanding contributions in village work but do not meet promotion criteria or have completed their village assignments, appropriate placement in suitable positions or the provision of training opportunities should be arranged so that they can continue to leverage their strengths. In addition, long-term village postings may lead to fatigue and exacerbate family tensions. It is therefore necessary to implement a rotation system for resident work team members and to arrange their work schedules and assignment cycles in a reasonable manner. The spatial allocation of resident work team members should also be optimized; based on local needs and resident work team members’ professional expertise, personnel should be deployed in a scientifically informed way to improve the efficiency and quality of village work. By stimulating work enthusiasm and ensuring that resident work team members have “aspirations, expectations and prospects,” their PSM and sense of responsibility can be further strengthened, enabling them to give full play to their distinctive advantages in the implementation of public policies and the advancement of the rural revitalization strategy.

Fourth, an organizational climate that encourages innovation and practical engagement should be actively fostered. It is crucial to provide resident work team members with continuous and precisely targeted support in terms of resources and capability development. Existing rigid training models should be reformed; practical approaches such as field-based “classrooms in the fields,” cross-regional study tours and training sessions delivered by professional managers and experts can rapidly enhance resident work team members’ practical skills. At the same time, a resource demand list system should be introduced, coordinated by higher-level organizations, to ensure that resident work team members are able not only to formulate project ideas but also to effectively connect with key resources such as policies, funding and technologies, thereby addressing the classic constraint of “having no material to work with.” Moreover, cultivating an organizational culture that combines strict standards with tolerance for well-intentioned mistakes and that rewards concrete performance is indispensable for mobilizing resident work team members’ intrinsic motivation. On the basis of scientifically designed assessment systems, a clearer orientation of “judging cadres by their practical achievements” should be established. Resident work team members should not only be encouraged to value substantive work outcomes, but also to listen closely to residents’ voices and to demonstrate responsibility and commitment in urgent, complex and high-risk tasks. For those who perform exceptionally well and achieve outstanding results, institutional bottlenecks in career development should be appropriately relaxed, and priority should be given to them in promotions, honors and awards, thereby forming a strong and sustained positive incentive mechanism.

### 6.2 Further discussion

To complement our empirical findings, we synthesize prior research, policy documents, and field insights to further elaborate on the relationships among job burnout, POS, and PSM. First, PSM may be subject to gradual erosion under stringent institutional conditions. It suggests that PSM cannot be assumed to be automatically or continuously sustained in the high-pressure task environment of grassroots governance. For grassroots public service workers who are chronically exposed to adverse work conditions, including role overload, excessive workloads, fragmented assessment requirements, and insufficient governance resources, job burnout may progressively deplete psychological resources, weaken role identification, and undermine self-efficacy, thereby attenuating the situated expression of PSM. Meanwhile, existing studies on PSM have predominantly examined positive antecedents, such as public values, organizational commitment, leadership support, mission identification, and perceived organizational support, thereby emphasizing the formation, activation, and reinforcement of PSM (Perry and Wise, 1990; Moynihan and Pandey, 2007; Chen and Lin, 2016). Departing from this dominant line of inquiry, the present study shifts analytical attention from the generative mechanisms of PSM to its potential erosive mechanisms. In this regard, the study extends the discussion of negative antecedents in PSM research and further clarifies the mechanism through which job burnout erodes PSM.

Second, PSM should not be understood as a stable trait that remains entirely invariant across contexts. This study does not deny the relatively stable value-oriented nature of PSM. Rather, it emphasizes that the situated expression of PSM, as well as its translation into public service behavior, is shaped by specific institutional environments and work contexts. Resident work team members are commonly regarded as a group with relatively high levels of PSM. In the context of China’s rural revitalization, their village-based work is also characterized by a salient political-task orientation. Nevertheless, even under top-down policy assignments, the maintenance of PSM cannot rely solely on political mobilization. Instead, it should be understood as embedded in a dynamic institutional cycle of “needs–motivation–behavior–new needs.” Management arrangements, support mechanisms, and incentive policies at different stages may influence individuals’ self-regulation, role cognition, and behavioral expectations, thereby causing their motivational levels to fluctuate across changing contexts. In the high-pressure setting of rural governance, individuals with high PSM often need to invest substantial self-regulatory resources to cope with complex administrative tasks, villagers’ demands, and multiple layers of assessment pressure. When organizations fail to replenish work resources in a timely manner, reasonably reduce task burdens, or provide effective support, individuals’ motivational levels may gradually decline as their psychological and work-related resources are depleted. Moreover, bureaucratic pressure, local interpersonal relations, resource constraints, and the complexity of governance tasks in rural China are all characterized by temporal and spatial variability. Consequently, the PSM of resident work team members cannot be maintained independently of concrete governance contexts; rather, it is continuously adjusted in response to external contextual factors such as task difficulty, public feedback, organizational support, and assessment orientation. Compared with approaches that conceptualize PSM primarily as a stable individual trait, a situated motivational perspective is better able to capture behavioral variations among resident work team members under different policy incentives and task pressures. It is also more consistent with the practical logic of relay-style governance and the dynamic adjustment of village-based work. By examining the situated expression of PSM among Chinese resident work team members, this study further clarifies how the stable trait component of PSM may vary in specific governance contexts and enriches the contextual applicability of PSM theory.

Third, the moderating effect of POS on the relationship between job burnout and PSM essentially reflects the stress-buffering function of POS. The findings indicate that when resident work team members encounter resource deficiencies, role conflicts, and task-related pressures, resource supplementation, problem-solving assistance, and value recognition from dispatching organizations and relevant governance systems can help compensate for work-related constraints, reduce internalized pressure, and slow the erosion of PSM caused by job burnout. This mechanism is conceptually distinct from the driving effect generated by political mobilization or the transmission of administrative pressure. Political mobilization and administrative pressure often compel grassroots actors to act by intensifying task urgency, responsibility constraints, and performance requirements. Although such mechanisms may increase task execution intensity in the short term, they do not necessarily provide long-term protection for individual motivation and may even further intensify the consumption of psychological resources among resident work team members. Accordingly, within the specific context of grassroots village-based governance in China, this study further distinguishes perceived organizational support from political mobilization, two concepts that have often been implicitly conflated. The POS emphasized in this study does not refer to administrative requirements, supervisory pressure, or political exhortation. Rather, it refers to the extent to which resident work team members perceive organizational recognition, care, value affirmation, and practical assistance when they encounter difficulties in village-level work. Its core dimensions include resource provision by dispatching organizations, work-related assistance, problem-solving support, and recognition of individual contributions. By distinguishing these two concepts at the operational level, this study provides a conceptual reference for the definition and measurement of related constructs in subsequent grassroots governance research and helps prevent the conflation of supportive organizational resources with pressure-oriented administrative mobilization.

Fourth, in the process of China’s rural revitalization, greater attention should be paid to the psychological mechanisms of individual policy implementers. On the one hand, the PSM of resident work team members should not be regarded as an inexhaustible moral resource, but rather as a motivational orientation that requires continuous institutional support and organizational reinforcement. On the other hand, the maintenance of PSM among resident work team members cannot be achieved solely through political mobilization or mission-based appeals. Instead, it depends largely on whether they perceive that their organizations recognize their contributions, support their task implementation, protect them from excessive accountability risks, and provide reasonable welfare guarantees and career development expectations. In other words, the sustainability of PSM is not merely driven by individual moral commitment; rather, it is institutionally embedded in specific arrangements of organizational support, resource provision, accountability protection, and career incentives. By approaching the issue from the psychological dimension of policy implementers and focusing on the critical historical transition from poverty alleviation to comprehensive rural revitalization, this study reveals the mechanism linking job burnout, POS, and PSM. It not only provides a theoretical explanation for the construction of resident work teams and the optimization of grassroots governance, but also offers policy implications for improving institutional design. In advancing rural revitalization and strengthening grassroots governance capacity, future policy efforts should further improve the organizational support system for resident work team members, optimize assessment and accountability mechanisms, enhance welfare guarantees and career development arrangements, and reduce unnecessary formalistic burdens. These measures can provide a more stable institutional foundation for the sustained maintenance and effective behavioral translation of grassroots public service motivation.

## 7 Limitations and future research

Despite its contributions, this study is subject to several limitations that suggest avenues for future research. First and foremost, several limitations regarding generalizability should be acknowledged. The sample of this study was drawn from resident work team members in the Wuling Mountains region of China, a region with distinctive socioeconomic conditions, rural governance challenges, and policy significance in the transition from poverty alleviation to rural revitalization. Therefore, the research findings need further verification when generalized to resident work team members in more developed regions, urban grassroots public employees, or public servants in different administrative systems. Second, beyond the scope of the sample, the underlying psychological mechanisms warrant further investigation. From the perspective of Conservation of Resources theory, job burnout may weaken the situated expression of PSM through resource-depleting psychological processes, including intensified role stress, reduced psychological empowerment and self-efficacy, and weakened role identification. However, these potential mediating mechanisms have not been directly measured. Although the theoretical logic of this study is grounded in the institutionalized stressors of resident work tasks, future research should adopt a cross-lagged panel design and scientifically measure these mediating mechanisms, so as to rigorously validate the dynamic motivational erosion process associated with job burnout. Third, given the individual-level design of the survey, this study does not further distinguish the potential effects of village-level contextual factors. In this regard, future research may adopt matched individual- and village-level data and employ multilevel approaches to examine how ecological and governance contexts shape the relationships among job burnout, POS, and PSM.

## Data Availability

The raw data supporting the conclusions of this article will be made available by the authors, without undue reservation.
